# Study on Economic Significance of Rare Earth Mineral Resources Development Based on Goal Programming and Few-Shot Learning

**DOI:** 10.1155/2022/7002249

**Published:** 2022-05-09

**Authors:** Shuxian Zhang

**Affiliations:** School of Earth Sciences and Resources, China University of Geosciences (Beijing), Beijing 100083, China

## Abstract

Rare earth is one of the most important strategic minerals in the world today. The wide application of new products and technologies in the global market has made the world's demand for rare earth resources grow rapidly. As an important basic resource of high technology, rare earth plays a significant role in national security and strategy. As rare earth mineral resources are irreplaceable in civil, military, and nuclear industries, they have become national strategic resources of various countries in the world. Although China's rare earth industry has occupied a leading position in the world, with the continuous expansion of the scale of the rare earth industry, the pollution problem of “three wastes” produced in the recovery of rare earth mineral resources is becoming more and more serious. From the perspective of resource endowment, China is rich in rare earth resources, but the declining trend of resource reserves is obvious, and the advantages and disadvantages of resources are more prominent. Based on the goal planning and few-shot learning, this paper studies the economic significance of rare earth resource development, aiming at solving some problems in the development of rare earth mineral resources in China, and thus promoting the scientific and healthy development of the rare earth industry.

## 1. Introduction

Rare earth elements have unique electronic structure, which makes them have excellent magnetic, optical, and electrical characteristics. They are widely used in metallurgical machinery, petrochemical industry, electronic information, energy and transportation, national defense and military industry, high-tech materials, and other industries [1]. As an important high-tech basic resource, rare earth plays an important role in national security and strategy. The most important feature of the raw material base developed so far in the world is that its structure is conducive to large or even super large deposits. China has basically formed a legal system for the protection and management of mineral resources guided by the Constitution and dominated by laws, administrative regulations and departmental rules, which provides a clear legal basis for the management of rare earth resources according to law [2]. Many experts believe that these deposits will be relocated according to the special characteristics of rare earth mineral composition [3]. Due to the progress of China's rare earth metallurgy technology and low cost, foreign rare earth smelting and separation enterprises have closed down one after another and the relevant research is gradually reduced [4]. Although China's rare earth industry has occupied a leading position in the world, with the continuous expansion of the scale of the rare earth industry, the pollution problem caused by the recycling of rare earth resources is becoming more and more serious [5]. This situation has affected the healthy, sustainable, and stable development of China's industrial system. It is urgent to develop an efficient and practical green mining and smelting process to solve the problem of environmental pollution caused by three wastes.

Rare earth is an indispensable strategic resource for countries all over the world to transform traditional industries and develop high-tech and cutting-edge national defense technology. We need to re-examine China's mineral resources legal system, explore the root causes of the problems from the theoretical basis and system design, and make up for the insufficient theoretical preparation and unreasonable system design [6]. Then, put forward the feasible countermeasures to improve China's legal system of mineral resources and then study the management of rare earth according to law [7]. China is not only a big country in rare earth resources but also a big country in rare earth production, export, and consumption in the world. Some countries and regions have listed it as a key element for the development of high-tech industries and a national strategic element, which is called an indispensable “industrial vitamin” [8]. From the perspective of resource endowment, China is rich in rare earth resources, but the declining trend of resource reserves is obvious, and the advantages and disadvantages of resources are more prominent [9]. Starting from the theoretical level, this paper abandons the theory that administrative license is the direct allocation of resources and insists that administrative license is only the franchise qualification granted by the government to the opposite party.

Heavy rare earth mineral raw materials mainly include phosphorus manganese ore, brown yttrium ore, ion adsorption rare earth ore, and uranium ore [10]. The rare earth industry is China's advantageous industry. The comprehensive management of the rare earth industry will greatly promote the healthy development of China's rare earth industry and better serve China's high-tech industry, new energy, aerospace, and national defense [11]. In the part of rare earth exploration license, closely follow the pace of China's geological exploration pattern reform, grasp the latest reform trends, put forward suggestions to speed up the establishment of mineral exploration financing market, and promote the exploration of rare earth and other strategic minerals [12]. As rare earth resources are irreplaceable in civil, military, and nuclear industries, they have become national strategic resources of countries all over the world [13]. Although China has obvious advantages in rare earth resources, due to lack of understanding, China's rare earth industry started late and the long-term development and management of rare earth resources are extensive [14]. In practice, the phenomenon of indiscriminate mining and blind competition among enterprises has become increasingly prominent, resulting in a large outflow of resources at low prices [15]. Based on the goal planning, this paper studies the economic significance of the development of rare earth resources, aiming to solve some problems in the development of rare earth resources in China, coordinate the development and utilization of rare earth resources with the ecological environment, and promote the scientific and healthy development of rare earth industry.

Section 2 analyzes the general situation of China's rare earth resources from two aspects: the characteristics of resource endowment and the characteristics of resource trade. Section 3 discusses the economic significance of rare earth resources through the goal planning method. Section 4 summarizes the content of the full text.

## 2. Overview of Rare Earth Resources in China

### 2.1. Characteristics of Resource Endowment

From the perspective of resource trade, the export-oriented and primary product-oriented trade structure has further weakened China's advantage of large quantity and variety of rare earth resources and intensified excessive competition in the rare earth industry. Between the upstream and downstream of the rare earth industry chain is a process of increasing value. At present, the profits of the rare earth industry are concentrated at the back end of its industry chain. Although rare, rare earth elements have not been known and used for a long time. It was not until the end of the 18th century that people discovered the existence of rare earth elements. Through research, people gradually realized its value and applied it to people's production and life. While China's rare earth is overexploited and its reserves continue to decline, foreign rare earth is being strategically protected and proven reserves are also increasing, leading to the gradual loss of China's rare earth reserve advantage [16]. Rare earth minerals in our country are mainly distributed in remote areas. A large number of small enterprises are engaged in rare earth resources development. The management mode is extensive and destructive mining, resulting in a large amount of rare earth resources loss and destruction [17]. Ultrahigh purity rare earth metals are the material basis for studying the intrinsic properties of rare earth and developing new rare earth materials. The purity of rare earth metals is also one of the key factors affecting and restricting the properties of rare earth functional materials.

**Figure 1:**
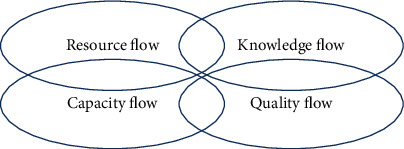
Extension Manufacturing Capability Unit model.

With the increasing demand for rare earth resources in China's high-speed economic growth, the export volume of rare earth is decreasing year by year, but the export volume of rare earth and other dominant minerals in China still occupies an important position in the world mineral trade. The rapid establishment, integration, reconfiguration, and dissolution of the Extension Manufacturing Capability Unit, as well as the rapid diffusion, rapid remodeling, and rapid transformation of manufacturing capability. There are four kinds of flows in the diffusion process of the Extension Manufacturing Capability Unit. The combined effect of these four streams enables large engineering equipment manufacturing systems to emerge rapidly expanding manufacturing capabilities in a short time.  Figure 1 is a model of the Extension Manufacturing Capability Unit.

**Figure 2:**
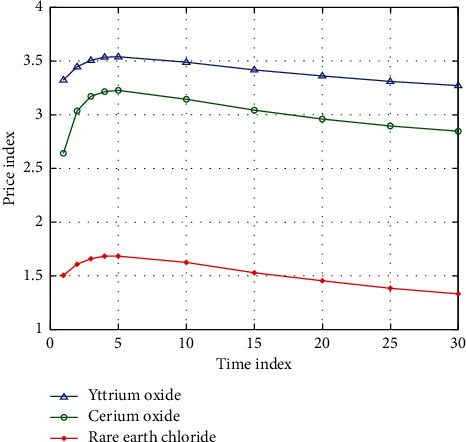
Changes in export prices of rare earth products.

The smuggling of rare earth resources in China is very serious, disrupting the order of the rare earth market, invalidating the national rare earth control policy, and failing to achieve industrial integration goals. Although rare earth resources are stored in large quantities in the world, due to the difficulty in industrial smelting and purification in practical applications, rare earth resources are still extremely rare. According to the geographical area, the geographical distribution of rare earth resources is broad and there is no monopoly of a certain region. Rare earth minerals are nonrenewable resources. Although major developed countries have been striving to develop alternatives, the harvests are minimal and some of the so-called alternatives are difficult to achieve the desired results in application. Therefore, rare earth resources are more precious [18]. The vast geographical distribution of rare earth resources determines that it is unrealistic to want to obtain a dominant say in rare earth resources for a long time by tightening supply ports. Compared with energy and basic metals, rare earthconsumption is very small and under the current visible application, the increment in various consumption fields is also limited. The strategic reserve system of resources should be implemented. Once the price of important mineral resources fluctuates abnormally and there is an interruption or shortage of supply, the strategic reserve resources can be put into the market to adjust the relationship between supply and demand in the market, thus greatly improving the right to speak on the price of mineral resources. Generally speaking, the price of rare earth resources has been low for a long time and the economic benefits are limited. At the same time, the environmental cost of rare earth resources development is high, which makes the overall benefits of rare earth resources export low.  Figure 2 shows the changes in export prices of rare earth products in China.

There are *n* indicators for evaluating the independent innovation capability of rare earth enterprises. Based on this part of the indicators, the independent innovation capability of enterprises is quantitatively divided into *T* grades, which are described as the following quantitative comprehensive evaluation matter element models:

(1) $$\begin{array}{c} Q\left(u_{ij}\right)=\sum_{i=1}^{n}\underset{1\leq j\leq m}{\text{Max}}\left\{g_{ij}\left(T\right)\right\}. \end{array}$$

The matter-element model formed by the comprehensive evaluation of the independent innovation ability and the allowable value range of each indicator is called the domain matter element.

(2) $$\begin{array}{c} y_{f-n_{m}}=\sum_{i=1,i\neq n}^{N}\sum_{l=1}^{M}\sqrt{p_{i_{l}}}h_{i,n_{m}}^{T}W_{i,i_{l}}s_{i_{l}}. \end{array}$$

For the enterprise to be evaluated, the data or analysis result obtained by the evaluation is represented by the matter element *P.*

(3) $$\begin{array}{c} P_{f-n_{m}}=\sum_{i=1,i\neq n}^{N}\sum_{l=1}^{M}p_{i_{l}}{\left\|h_{i,n_{m}}^{T}W_{i,i_{l}}\right\|}_{2}^{2}. \end{array}$$

### 2.2. Characteristics of Resource Trade

A large number of foreign-funded enterprises have evaded the export quota of rare earth resources in China by investing in factories and other ways and have carried out predatory mining. Only simple processing can be exported, resulting in a large amount of waste of rare earth resources. The abundance of rare earth elements in the earth's crust is not barren, even relatively rich, and the abundance of some elements is higher than that of common metals such as copper and zinc. Rare earth elements are widely distributed in the Earth's crust and there are many mineral occurrences. However, there are no more than 30 large companies that control rare earth resources in the world. If you master the trends of these 30 companies, you can directly understand the global supply and demand of rare earth and the industrial chain [19]. The protection and management of rare earth resources in China have been gradually implemented with the breakthrough of rare earth application technology and the development of the rare earth industry. We should strengthen macrocontrol and give full play to the leading role of the government. The establishment of a rare earth industry enterprise alliance to control the price and usage of concentrate and to control the price and sales of primary products. Formulate relevant policies to encourage rare earth enterprises to go abroad and participate in or take control of the development of foreign rare earth resources, so that China's rare earth industry will truly go international.

**Figure 3:**
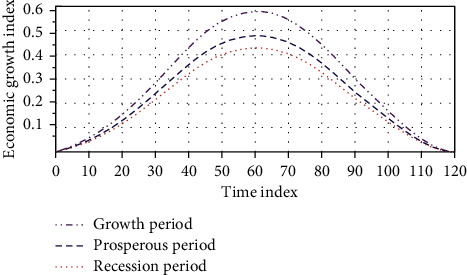
General law of resource-based urban development.

Although China prohibits the export of rare earth raw ores, the economic benefits brought to China by the export structure dominated by rare earth separation products are relatively low, and rare earth smelting separation will cause serious pollution. From the beginning, two mechanisms with the nature of compensation and assistance were proposed, i.e., compensation mechanism for resource development and the assistance mechanism for declining industries. Sustainable development is based on natural assets and coordinated with environmental carrying capacity. Sustainable development aims at providing quality of life and is compatible with social progress. Research in any discipline or field should be supported by scientific methods that meet the needs of its research objects and contents and are determined by its research purposes. At the same time, any scientific research method is not exclusive but universal.  Figure 3 is a schematic illustration of the general law of the development of resource-based cities.

The relevant data are substituted into the system to run, and the original model is repeatedly corrected to obtain a series of fitting indexes of the corrected model. When the significance level is low, the customer management ability has a relatively large impact on the marketing performance. However, the other two subindicators market learning ability and marketing promotion ability have a significant impact on marketing performance but to a lesser extent. In Table 1, the structural parameter estimation and significance test of performance indicators is shown.

**Figure 4:**
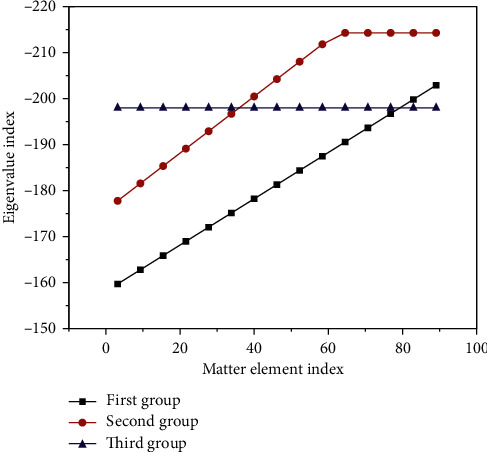
Relationship between eigenvalue and matter element.

Although the absolute amount of rare earth is large, the content is low and the average abundance in the Earth's crust is only 200 ppm, and the distribution is very uneven. In the matter element, A is an evaluation feature, that is, an indicator. Some feature values can be calculated by the system, and the feature values corresponding to each feature are shown in Table 2.  Figure 4 shows the relationship between eigenvalues and matter elements in three sets of experimental data.

China's political system, economic system, and the development of the rare earth industry determine the legal system and policy measures for the management of rare earth resources. At present, almost all oxidizing roasting-hydrochloric acid leaching method is used in the industry. The rare earth ore is oxidized and calcined, and the bastnasite is decomposed to form rare earth oxide, rare earth fluoride, or rare earth oxyfluoride which is soluble in hydrochloric acid [20]. Rare earth elements are mostly in the form of oxides or symbiotic forms of oxoacid minerals, and the degree of enrichment is low. There are not many deposits with mining value at the current technical level, so rare earth resources have been proven in the world. The amount of exploitation is scarce. The in situ leaching process does not requires excavation of ore bodies, has little impact on the ecological environment, and has high resource utilization [21]. However, the in situ leaching process developed in the early stage is only applicable to mines with intact ore bodies. For mines with complex geological conditions, leaching liquid leakage often occurs, resulting in a significant reduction in rare earth recovery.

## 3. Economic Significance of Rare Earth Resources

Whether the political and legal system of rare earth resources protection and management can adapt to the development requirements of the rare earth resources industry determines the success or failure of China's rare earth resources strategy. Rare earth enterprises should develop new products to meet the domestic demand for rare earth consumption. Rare earth, as a special mineral resource, is greatly different from other mineral resources in terms of resource structure, resource use, and spatial distribution. In view of the problems of high consumption of chemical materials, the low comprehensive utilization rate of resources and serious pollution of the three wastes in the rare earth smelting and separation process, various research institutions, and enterprises have developed a series of efficient, clean, and environmentally friendly smelting and separation processes. In order to reduce dependence on foreign rare earth resources and prevent being controlled by others, some developed countries have begun to develop substitutes for rare earth resources. Judging from the current scientific and technological achievements, although some scientific and technological progress has been made, the results are not good. In order to better protect rare earth resources, China's established rare earth strategic resource reserve system should be implemented and perfected as soon as possible [22]. The unified export of rare earth products has also better maintained the reasonable price of rare earth, but there are also unreasonable problems, such as neglecting the management and protection of light rare earth and failing to adjust the relationship between rare earth development and environmental protection. Rare earth enterprises should seize the market opportunity, follow closely the market demand, and strive to develop the domestic market.

**Figure 5:**
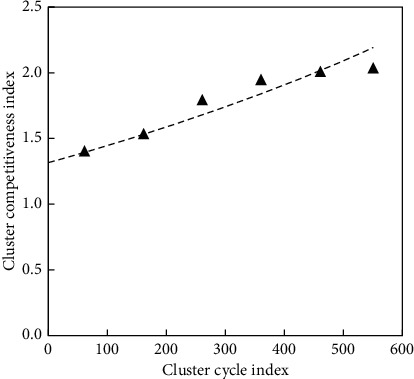
Trends in the competitiveness of resource-based clusters.

Under the current economic situation, small mining companies should adhere to the principle of large and small, superior and inferior, and follow the laws of the market economy according to the natural occurrence of resources. The social network formed by resource-based urban clusters is a complex and dynamic network. A social implementation mechanism such as a cluster network can establish a cluster trust mechanism to improve the resource environment. At different stages, the strength of urban cluster competitiveness and the specific performance of urban cluster competitiveness at different stages are shown in Figure 5.

**Figure 6:**
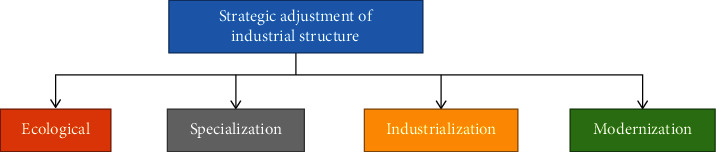
Strategic adjustment structure of the resource-based urban industrial structure.

The market learning ability has no significant influence on the accounting effect and brand assets. It actively promotes the construction of modern characteristic resource-based industries and the modernization of production methods, production methods, and production concepts. Speed up the transformation of industrial development mode, save natural resources and production factors, optimize the economic structure of resource-based cities, and realize sustainable development.  Figure 6 is a schematic structure for strategic adjustment and guidance of industrial structure in resource-based cities.

The mode of unified purchase and sales of rare earth products was severely impacted by the market economy and withdrew from the historical stage. Therefore, the state's policy of controlling rare earth mainly focuses on mining and export. Through orderly mining, effective protection of resources is realized, and at the same time, local regional economic transformation and industrial upgrading are also supported. China's failure to enact a superior mineral protection law in time to meet the requirements of the development of the market economy has put the country's protection of rare earth resources into a dilemma. Most of the rare earth minerals in the world contain only a few rare earth elements, which are incomplete. The rest of the rare earth elements that do not exist still depend on imports [23]. China Nonferrous Engineering Design and Research Institute and Rare Earth Material Factory have improved the low-temperature roasting process to solve the problem of wall formation. Continuous low-temperature dynamic roasting can be realized by using low-temperature curing technology. Although the abundance of rare earth elements in the Earth's crust is not poor or even higher than that of some common metals, due to the technical conditions, some rare earth ores have low mining value and high cost, which are difficult to meet the mining standards and cannot be applied by human beings.

**Figure 7:**
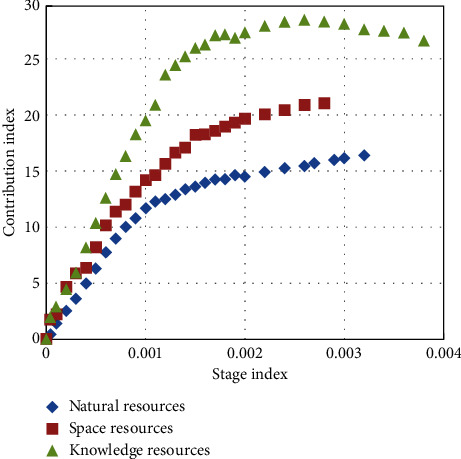
Different resources contribute to different stages of economic development.

No resource can function independently as a factor of production. Only when natural resources are combined with social resources can they be transformed into productive forces with use value and value. Rare earth enterprises should base themselves on the domestic market and expand domestic demand.  Figure 7 shows the contribution of different resources at different stages of economic development.

After the establishment of the comprehensive evaluation of the material-element model of independent innovation capability, it is necessary to evaluate the index value of each characteristic of the independent innovation capability of the enterprise and the approximation degree of each level. Applying the proximity of classical domain matter elements in the following extension set:

(4) $$\begin{array}{c} {\text{cell}}_{ps-1}=\underset{n}{\arg\;\max}\left(\sum_{m=1}^{M}P_{f-n_{m}}\right). \end{array}$$

The extension distance is

(5) $$\begin{array}{c} {\text{cell}}_{ps-n_{s1}}=\underset{i,i\neq {\text{cell}}_{ps-1},\ldots ,{\text{cell}}_{ps-n_{s}}}{\arg\;\min}\left(\sum_{m=1}^{M}\sum_{l=1}^{M}p_{i_{l}}{\left\|h_{i,ps-1_{m}}^{T}W_{i,i_{l}}\right\|}_{2}^{2}\right). \end{array}$$

Calculate the degree of association of the company to be evaluated with the weight coefficient of each feature.

(6) $$\begin{array}{c} R_{n_{i}}^{C}=\;\log_{2}\left(1+\frac{p_{\text{mac},n_{i}}{\left\|h_{\text{mac},n_{i}}^{T}W_{\text{mac},n_{i}}\right\|}_{2}^{2}}{\sigma^{2}}\right). \end{array}$$

**Figure 8:**
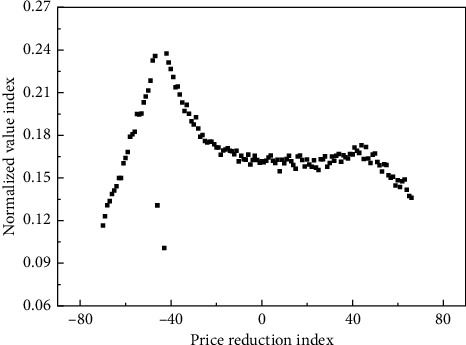
Relationship between normalized value and price reduction.

Through the deep processing of rare earth resources and the extension of the industrial chain, the pace of development of rare earth resources from upstream primary raw materials to deep processed products, high-tech new materials, functional components, and end-use products will be accelerated. After the various influencing factors have been uniformly measured to the four-point system, in order to better study the impact of price competition on various factors, the argumentation part adopts the idea of normalization of price competition. The price competition is divided into five sections, each of which has a length of 20%. The specific division is shown in Table 3. The relationship between the normalized value and the price reduction range is shown in Figure 8.

Sustainability is a measure of the sustainability criteria for resource-based urban transformation systems and the development of subsystems. Using the development rate of each subsystem as a measure, the following can be obtained:

(7) $$\begin{array}{c} Q\left(u_{ij}\right)=\sum_{i=1}^{n}\underset{1\leq j\leq m}{\text{Max}}\left\{g_{ij}\left(T\right)\right\}. \end{array}$$

Using the fuzzy membership function method to obtain the following:

(8) $$\begin{array}{c} y_{f-n_{m}}=\sum_{i=1,i\neq n}^{N}\sum_{l=1}^{M}\sqrt{p_{i_{l}}}h_{i,n_{m}}^{T}W_{i,i_{l}}s_{i_{l}}. \end{array}$$

Calculate the trend degree as follows:

(9) $$\begin{array}{c} P_{f-n_{m}}=\sum_{i=1,i\neq n}^{N}\sum_{l=1}^{M}p_{i_{l}}{\left\|h_{i,n_{m}}^{T}W_{i,i_{l}}\right\|}_{2}^{2}. \end{array}$$

In the impact on the other three indicators, although there is a certain degree of positive impact, but the impact is less than the customer management ability. But it is very close, so its impact on brand equity can not be ignored. The impact of marketing capabilities on customer value and product innovation is not significant, and the positive impacts on the other three indicators are significant. Similarly, the factor load of the corresponding indicators is also less than the factor load associated with the customer. The structural parameter estimation and significance test of the impact of marketing subcategories on marketing performance subindicators are shown in Table 4.

The selection of resource-based city indicators should cover the content of the evaluation as much as possible and comprehensively reflect all aspects of the city's sustainability capacity. The correlation value of each level indicator is shown in Table 5.

To measure the degree of price competition between a company's homogeneous product and the market average price, you can simply write the price competition between a single company's product pricing and the market average price as follows:

(10) $$\begin{array}{c} c_{1}\left(t\right)=c_{11}+\left(c_{1T}-c_{11}\right)\frac{t}{T},\\ c_{2}\left(t\right)=c_{21}+\left(c_{2T}-c_{21}\right)\frac{t}{T}. \end{array}$$

Initialize, calculate the connection weight and threshold, and assign any value.

(11) $$\begin{array}{c} I_{i}={\left[\sum_{j=1}^{p}\omega_{j}^{m}y_{ij}^{m}\right]}^{\left(1/m\right)}. \end{array}$$

Provide input samples and expected output to obtain the following equation:

(12) $$\begin{array}{c} I_{\omega }\ddot{\delta }=F_{r}d-K_{\omega }\dot{\delta }-C_{\omega }\delta -K_{1}e\delta . \end{array}$$

Calculate the output of each unit in the hidden layer.

(13) $$\begin{array}{c} T=\frac{M}{R}\\ =\frac{i\eta_{e}M_{e}}{r}. \end{array}$$

Enterprises need to improve their independent innovation ability and promote the upgrading of industrial structures. Strengthen the research on the development strategy of the rare earth industry and formulate policies and measures to further promote the development of rare earth technology and industry. Rare earth minerals are China's dominant mineral resources. In the early stage of reform and development, it is historically inevitable to obtain considerable economic income by supplying a large amount of rare earth products to the international rare earth market. Compared with the world's large multinational enterprises, China's enterprises in related industries are not strong in independent innovation, market competitiveness, and ability to resist international market risks. Cultivating a number of large enterprises with strong comprehensive strength is an important goal. Heavy and light rare earth products are sharply divided, and the proportion of heavy rare earth products in the mines that have been put into production is relatively low, so they cannot impact the medium and heavy rare earth market. China will continue to maintain its leading position in this field [24].

**Figure 9:**
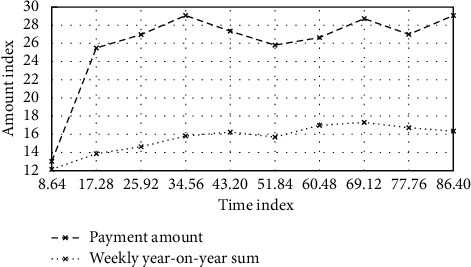
Preview of the transaction overview.

Rare earth resources are widely used in modern industrial development. Rare earth elements play a key role in some aspects. In the overall sales analysis, it is necessary to specifically analyze the customer unit price, year-on-year month-on-month changes, and other links. A preview of the transaction is shown in Figure 9.

China needs to fulfill relevant international commitments and obligations. The supply of rare earth products cannot be controlled by administrative means. Even through the means of environmental protection, its supervision system and law enforcement methods are not perfect and lack of supporting measures. The strategic value of rare earth resources is outstanding, and its supply directly affects the national defense industry, national competitiveness, and even the economic security of the entire country. The development of the rare earth resource industry not only provides a large number of jobs but also solves the problem of insufficient supply of mineral products in rapid economic development. In order to balance the interests of the central, local, and enterprise and properly handle the relationship between different regions and upstream and downstream industries, it is recommended to reconfigure mining licenses for state-led enterprises on the basis of the joint of national large-scale rare earth enterprise groups and resource regions [25]. We must first clearly understand the problems of China's rare earth resources in mining, processing, and export trade and find out the reasons through careful analysis and research, and then we can propose solutions to the problems. In order to enable China's rare earth industry to develop healthily and sustainably, it is recommended to increase investment in science and technology and environmental protection, strengthen research on efficient and clean production processes, industrial development and promotion, and strictly implement the “Rare Earth Industrial Pollutant Emission Standards.” You can choose several key links for key management to effectively manage the valuable rare earth resources that protect the country.

## 4. Conclusions

Rare earth minerals are the strategic resources of the country. Therefore, the distribution and use of rare earth mining rights should not be allowed to be bought and sold arbitrarily as ordinary metal mining rights, so as to realize the effective control of rare earth strategic resources by the country. At a time when the socialist market economy system is gradually improving, the reimplementation of comprehensive planning and control of rare earth and other advantageous mineral resources no longer meets the fundamental requirements of the market economy era but also violates the basic development trend of mining development and operation mechanism and management system. While rare earth development benefits mankind, the accompanying problems of resources and environment are becoming increasingly prominent. With the in-depth development of economic globalization, China's international exchanges and cooperation in the field of rare earth resources are increasing day by day. At the system level, different legal procedures should be applied to the grant of rare earth development and management rights and the assignment of rare earth mining rights, respectively, to clarify the differences between the franchise rights and property rights. Mining rights can be taken as state-owned capital and included in the asset management scope of central enterprises. The exploration right should be granted to large enterprise groups on a regional, limited, and limited basis to avoid the loss of new rare earth resources. It is suggested that departments of land and resources at all levels should clean up and rectify their registration in the name of polymetallic minerals, actually demarcate rare earth mineral resources, and re-examine the exploration licenses and mining licenses that have been issued in rare earth mineral resources areas. In terms of process innovation management, China should attach importance to the nonrenewable nature of rare earth resources and increase the capital investment for rare earth deep processing and process innovation as soon as possible.

## Figures and Tables

**Table 1:** Performance indicator structure parameter estimation and significance test.

Path description	*t* value	Path coefficient
Market learning ability ⟶ influence performance	3.46	1.57
Marketing ability ⟶ marketing performance	3.24	1.49
Customer management capabilities ⟶ marketing performance	4.37	1.45

**Table 2:** Characteristic values corresponding to each feature.

Features	A1	A2	A3	A4	A5	A6	A7	A8	A9
Characteristic value	75.6	88.5	87.3	95.2	74.8	74.7	86.2	91.7	76.5

**Table 3:** Price competition degree division.

Degree of decrease (%)	0–20	20–40	40–60	60–80	80–100
Normalized value	0.2	0.4	0.6	0.8	1

**Table 4:** Significant test of the impact of marketing subcategories on marketing performance subindicators.

	Value of customer	Competitive result	Brand equity	Product innovation
Market promotion ability	0.76	0.59	0.64	0.69
Customer management ability	0.37	0.44	0.49	0.51
Market learning ability	0.39	0.32	0.47	0.45

**Table 5:** Value of the correlation function of the evaluation level.

Grade indicator	1	2	3	4
S1	0.386	0.556	0.742	0.457
S2	0.943	0.796	0.297	0.362
S3	0.348	0.653	1.569	0.794

## Data Availability

The data used to support the findings of this study are available from the author upon request.
